# Crystal Chemistry and Antibacterial Properties of Cupriferous Hydroxyapatite

**DOI:** 10.3390/ma12111814

**Published:** 2019-06-04

**Authors:** Arjak Bhattacharjee, Yanan Fang, Thomas J. N. Hooper, Nicole L. Kelly, Disha Gupta, Kantesh Balani, Indranil Manna, Tom Baikie, Peter T. Bishop, Timothy J. White, John V. Hanna

**Affiliations:** 1 Department of Materials Science and Engineering, Indian Institute of Technology, Kanpur 208016, India; amarchalarpath@gmail.com (A.B.); kbalani@iitk.ac.in (K.B.); 2 School of Materials Science and Engineering, Nanyang Technological University, Nanyang Avenue, Singapore 639798, Singapore; YNFang@ntu.edu.sg (Y.F.); thooper@ntu.edu.sg (T.J.N.H.); DISHA001@e.ntu.edu.sg (D.G.); tjwhite@ntu.edu.sg (T.J.W.); 3 Department of Physics, University of Warwick, Coventry CV4 7AL, UK; N.Kelly.1@warwick.ac.uk; 4 Department of Metallurgical and Materials Engineering, Indian Institute of Technology, Kharagpur 721302, India; imanna@metal.iitkgp.ernet.in; 5 Energy Research Institute @ NTU (ERI@N), Research Technoplaza, Nanyang Technological University, Nanyang Drive, Singapore 637553, Singapore; TBaikie@ntu.edu.sg; 6 Johnson Matthey Technology Centre, Blounts Court Rd., Sonning Common, Reading RG4 9NH, UK; peter.bishop@matthey.com

**Keywords:** hydroxyapatite, copper doping, copper oxidation state, heat treatment, antibacterial efficacy, materials characterisation

## Abstract

Copper-doped hydroxyapatite (HA) of nominal composition Ca_10_(PO_4_)_6_[Cu_x_(OH)_2-2x_O_x_] (0.0 ≤ x ≤ 0.8) was prepared by solid-state and wet chemical processing to explore the impact of the synthesis route and mode of crystal chemical incorporation of copper on the antibacterial efficacy against *Escherichia coli* (*E. coli*) and *Staphylococcus aureus* (*S. aureus*) strains. Apatites prepared by solid-state reaction showed unit cell volume dilation from 527.17 Å^3^ for copper-free HA to 533.31 Å^3^ for material of the putative composition Ca_10_(PO_4_)_6_[Cu_0.8_(OH)_0.4_O_0.8_] consistent with Cu^+^ insertion into the [001] hydroxyapatite channel. This was less pronounced (528.30 Å^3^ to 529.3 Å^3^) in the corresponding wet chemical synthesised products, suggesting less complete Cu tunnel incorporation and partial tenancy of Cu in place of calcium. X-ray absorption spectroscopy suggests fast quenching is necessary to prevent oxidation of Cu^+^ to Cu^2+^. Raman spectroscopy revealed an absorption band at 630 cm^−1^ characteristic of symmetric O-Cu^+^-O units tenanted in the apatite channel while solid-state ^31^P magic-angle-spinning nuclear magnetic resonance (MAS NMR) supported a vacancy-Cu^+^ substitution model within the apatite channel. The copper doping strategy increases antibacterial efficiency by 25% to 55% compared to undoped HA, with the finer particle sizes and greater specific surface areas of the wet chemical material demonstrating superior efficacy.

## 1. Introduction

**Figure 1:**
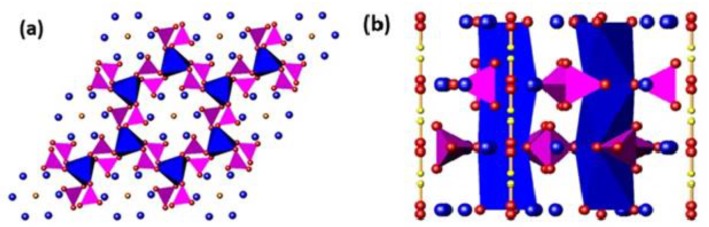
A polyhedral representation of the HA (Ca_10_(PO_4_)_6_(OH)_2_) structure projected along the (**a**) [001], and (**b**) [100] directions. The [001] projection highlights the PO4 tetrahedra (purple) corner-shared to CaIO6 metaprisms (blue). The channel OH− units are surrounded by CaII cations (blue spheres). The [100] projection emphasizes the linear disposition of OH^−^ units in the apatite channel (red and yellow spheres).

The crystallochemical formula of apatite is [A^I^_4_A^II^_6_](MO_4_)_6_[X_2_], where A is a larger cation (especially alkali, alkaline-earth, lanthanide), M a smaller cation, and X an anion [1,2,3]. These structural elements are arranged as an [A^I^_4_](MO_4_)_6_ framework surrounding an [A^II^_6_][X_2_] channel (Figure 1). Apatites can serve as pigments, catalysts, energy materials, bioceramics and solids for the remediation of hazardous waste with the flexibility to accept a multitude of cation and anion substitutions, thus modifying its physical, chemical and biological properties [4,5,6,7,8,9]. For the tenancy of first-row transition metals including Cu, Zn, and Fe, size (ionic radius) and oxidation state are the primary determinants of the mode of incorporation, with the heat treatment regime during synthesis also potentially modifying the site substitution profile [4,10,11,12,13].

Calcium hydroxyapatite (HA, ideal nominal stoichiometry Ca_10_(PO_4_)_6_(OH)_2_) is conventionally assigned hexagonal *P*6_3_*/m* symmetry with a Ca/P ratio of 1.67, similar to human bones and teeth, rendering it suitable for orthopaedic, dental and maxillofacial repairs [14,15]. According to Hoover et al. [16] and Weinstein et al. [17], musculoskeletal diseases and assaults are the second most prevalent cause of disability, affecting over 1.7 billion people worldwide. Therefore, HA-based composites are candidates for surgical bone implants. However, post-surgery periprosthetic infections arising from biofilm growth on implant surfaces remain a significant risk addressed by local antibiotic treatment. However, the well-regulated spatial and temporal delivery of drugs to prosthetic infections is challenging [18,19,20]. One solution is to use prostheses with inherent antibacterial properties. The healing capacity of Cu has been known from antiquity as described in ancient Indian, Egyptian and European texts [21,22,23], and more recently, Cu-doped HA has proven an effective treatment against gram-positive and gram-negative bacteria. Shanmugam et al. [24] reported the antibacterial efficiency of HA improved from 25% to 85% through the presumed partial displacement of Ca by Cu^2+^. Nonetheless, reports of the inclusion of Cu^+^ and/or Cu^2+^ in HA are sometimes conjectural, and studies of prosthetic applications usually do not consider crystal chemistry rigorously.

This study describes the incorporation of monovalent copper in HA of a nominal composition of Ca_10_(PO_4_)_6_[Cu_x_(OH)_2-2x_O_x_] (0 ≤ x ≤ 0.8 at increments of 0.2), where Cu^+^ notionally resides in the apatite channel. The Cu-doped HA systems were prepared by solid-state and wet chemical syntheses to investigate the crystal chemistry and antibacterial efficacy. The effect of the Cu oxidation state on *Escherichia coli* (*E. coli*) and *Staphylococcus aureus* (*S. aureus*) mortality and the implications for the deployment of Cu-doped HA as a bio-implant material for musculoskeletal surgery were considered.

## 2. Materials and Methods

### 2.1. Synthesis

**Figure A1:**
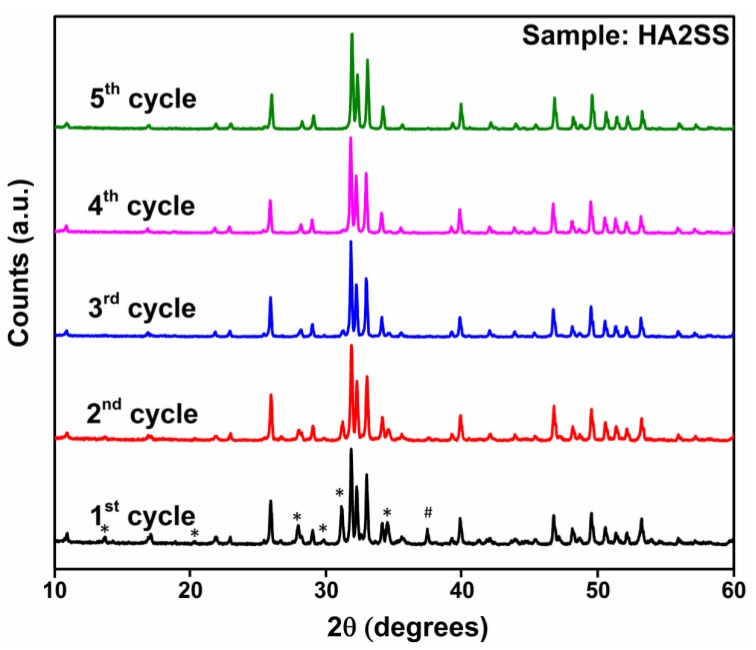
PXRD data showing the phase composition of sample HA2SS after successive sintering/quench cycles. The impurities observed are *β*-TCP (identified with *) and CaO (identified with #).

Solid-state and wet chemical methods were used to synthesise Cu-doped HA [1,12,25]. In the former, stoichiometric proportions of CaCO_3_ (ultrapure, Sigma-Aldrich) and (NH_4_)_2_HPO_4_ (ultrapure, Sigma Aldrich, Milwaukee, USA) were manually homogenised in an agate mortar for 30 minutes. The mixture was air-sintered (1100 °C soak/18 h/5 °C/min) and then air-quenched to produce HA. It has been demonstrated in earlier studies that rapid quenching could fix the Cu^+^ oxidation state without the need to resort to neutral or reducing atmospheres to prevent oxidation to Cu^2+^ [4,12,25]. The grinding, heating and quenching process was repeated until the sample was single phase by powder X-ray diffraction (PXRD) (see Figure A1 in Appendix A demonstrating this method). For the cupriferous analogues, copper was introduced as CuO (ultrapure, Merck, Darmstadt, Germany). As reported herein, the cuprous rather than cupric species was dominant after synthesis and incorporated in HA according to the substitution 2OH^−^ → Cu^+^ + O^2−^, while maintaining a Ca/P ratio of 1.67. The putative product compositions were Ca_10_(PO_4_)_6_[Cu_x_(OH)_2-2x_O_x_] (0.0 ≤ x ≤ 0.8) (Table 1). These apatites were supplemented by a single composition Ca_10_(PO_4_)_6_[Cu_0.6_(OH)_0.8_O_0.6_] (x = 0.6, HA6SS1) slowly cooled from 1100 °C for examination by X-ray absorption spectroscopy (XAS) to study the influence of quenching on the Cu oxidation state.

For soft chemical syntheses, 100 mL of 0.5 M Ca(NO_3_)_2_∙4H_2_O (ultrapure, Merck, Darmstadt, Germany) was mixed dropwise with 100 mL of 0.3 M (NH_4_)_2_HPO_4_ (ultrapure, Sigma-Aldrich, Milwaukee, USA) at a rate of 2 mL/min, while maintaining a pH of 10–12 with NH_4_OH (25% NH_3_) [26,27]. The reaction continued for 2 h at 95–100 °C under magnetic stirring. The products were collected by centrifugation (6 mins) at ambient temperature (24 ± 4 °C), washed three times with deionised water and then dried (120 °C for 24 h). Copper was incorporated as stoichiometric quantities of Cu(NO_3_)_2_∙3H_2_O (ultrapure, Sigma Aldrich, Milwaukee, USA) to the Ca(NO_3_)_2_∙4H_2_O solution and then dried, sintered and quenched as for the solid state route (1100 °C soak/18h/5 °C/min/air quench). Two additional Cu-free HA samples (HAWCM700°C (700 °C/18 h/5 °C/min) and HAWCM1100°C (1100 °C/18 h/5 °C/min)) were prepared as standards for solid-state ^31^P magic-angle-spinning nuclear magnetic resonance (MAS NMR) by sintering and then slow cooling in the powered-off furnace. 

### 2.2. Phase Confirmation and Characterisation

Powder X-ray diffraction (PXRD) patterns were collected using a Bruker D8 Advance instrument (Bruker, Billerica, USA (Bragg-Brentano geometry)) equipped with a Cu Kα (1.5406 Å) X-ray tube operating at 40 kV and 40 mA with data accumulated at a step size of 0.02° 2θ and dwell time of 2 s per step. To determine unit cell parameters, Rietveld analyses were carried out from 20° to 80° 2θ with the TOPAS V3 software (Bruker, Billerica, USA) package [28], assuming a *P*6_3_/*m* symmetry for Cu-doped HA [1,3]. A pseudo-Voigt peak shape function modelled the Bragg reflections and a four-coefficient Chebychev polynomial, and a 1/x profile background, a zero error, scale factors and lattice dimensions were refined sequentially. The incorporation of Cu was verified by monitoring the progressive variation of the lattice parameters. Secondary phases, particularly CuO, α/β-tricalcium phosphate (TCP, Ca_3_(PO_4_)_2_) and CaO were detected in some samples. The microstructure and microchemistry were analysed by field emission scanning electron microscopy (FESEM, Carl Zeiss SMT AG, Oberkochen, Germany,) and energy dispersive X-ray spectroscopy (EDS) using a Cilas-1064 attachment (Oxford instruments, Abingdon, Oxfordshire, UK). Particle sizing of the resultant undoped and Cu-doped biomaterials was undertaken by laser scattering using ImagJ (2018) software (National Institute of Health & university of Wisconsin, USA), where 10 different particle sizes are measured with a calculated average ± standard deviation value being reported. Fourier transform infrared spectroscopy (FTIR, Perkin-Elmer Spectrum 2000) (Perkin Elmer, Inc., Waltham, USA) was performed by the KBr pellet method over a spectral range of 400 to 4000 cm^−1^ (spectral resolution of 4 cm^−1^ and accumulated for 32 scans). To confirm O-Cu-O bonding, micro-Raman spectroscopy was performed (Princeton Instruments, STR Raman, TE-PMT detector) in backscattering mode with a Nd YAG green laser (532 nm/12.5 mW), a 50X objective and a spatial resolution of 0.5 cm^−1^.

A preliminary assessment of Cu valence and local coordination was conducted using the X-ray absorption fine structure for catalysis (XAFC) beamline at the Singapore Synchrotron Light Source (SSLS). All X-ray absorption spectra (XAS) were measured at a ring energy of 0.7 GeV and current of ~200 mA. A Si (111) crystal monochromator tuned the X-ray energy, and all data were acquired in transmission mode at ambient temperature from pressed powder pellets (10 mm diameter) from 8850 to 9100 eV bracketing the Cu K absorption edge. Comparisons were made against Cu_2_O and CuO standards. Data were analysed with Athena and Artemis features included in the Demeter software package (V 0.0.25) (National Institute of Standards and Technology, Gaithersburg, USA) with Artemis fittings performed with a k-weight = 1, 2, 3; k range = 3–12.5 and an R range = 1–3 Å.

Solid-state ^31^P magic-angle-spinning nuclear magnetic resonance (MAS NMR) data were acquired at 14.1 T using Bruker Avance II+-600 and Bruker HD-600 spectrometers (Bruker BioSpin, Rheinstetten, Germany) operating at a ^31^P Larmor frequency of *ν*_0_(^31^P) = 242.96 MHz. These measurements were performed using either Bruker 1.9 mm or 2.5 mm HXY MAS probes which enabled MAS frequencies (*ν*_r_) of 12–15 kHz. All ^31^P MAS NMR data were externally referenced to the 85% H_3_PO_4_ (IUPAC standard reference) via a solid ammonium dihydrogen phosphate ((NH_4_)H_2_PO_4_) secondary reference which is observed at *δ*_iso_ = 0.99 ppm. These data were acquired using a single pulse experiment which utilised high power ^1^H decoupling during acquisition. A *π*/2 pulse length of 2.6 *μ*s was calibrated using solid (NH_4_)H_2_PO_4_, and each measurement employed an excitation flip angle of *π*/4 (pulse length of 1.3 *μ*s) and a recycle delay of 180 s. The ^1^H decoupling field strength during the acquisition was 100 kHz. All simulation and deconvolution of the ^31^P MAS NMR data was undertaken using the DMFit software programme [29].

### 2.3. Antibacterial Efficacy

Gram negative *E. coli* (MCC2079) bacteria and gram positive *S. aureus* (MCC2043) bacteria were obtained from the National Centre of Cell Science, Pune, India, cultured in a Luria broth (LB) medium and incubated overnight at 37 °C. A single colony of bacteria was isolated by streaking the culture on an agar plate following 24 h incubation in fresh medium. Bacterial seeding was performed using this suspension with a bacterial optical density (OD) of 0.1 (at 600 nm, 10^8^ CFU/mL of medium). Apatite powders (1.5 mg) were mixed with 1 mL of LB containing 5 x 10^8^ CFU/mL bacterial cells of *E.coli*, which was incubated for 4 h at 37 °C. A similar method was followed for *S. aureus*, only with the control sample (undoped HA), HA6WCM and HA6SS (composition chosen after analysing the results against *E. coli*). Bacterial assays were performed using 3-(4,5-dimethylthiazol-2-yl)-2,5-diphenyl tetrazolium bromide (MTT) [30], and the optical absorbance of the solution (at 540 nm) was measured using a BioTek ELx800 micro-plate reader (Labx, San Diego, USA). Post-exposure, the counts (percentage microorganism reduction, R%) of viable microorganisms on both the solid-state and wet chemical materials were compared with pure HA (positive control sample). The experiments were triple replicated, and an average of the results was taken. The statistical analysis of all data was performed by using a t-test with 95% confidence level.

## 3. Results and Discussion

### 3.1. Phase Assemblages

**Figure 2:**
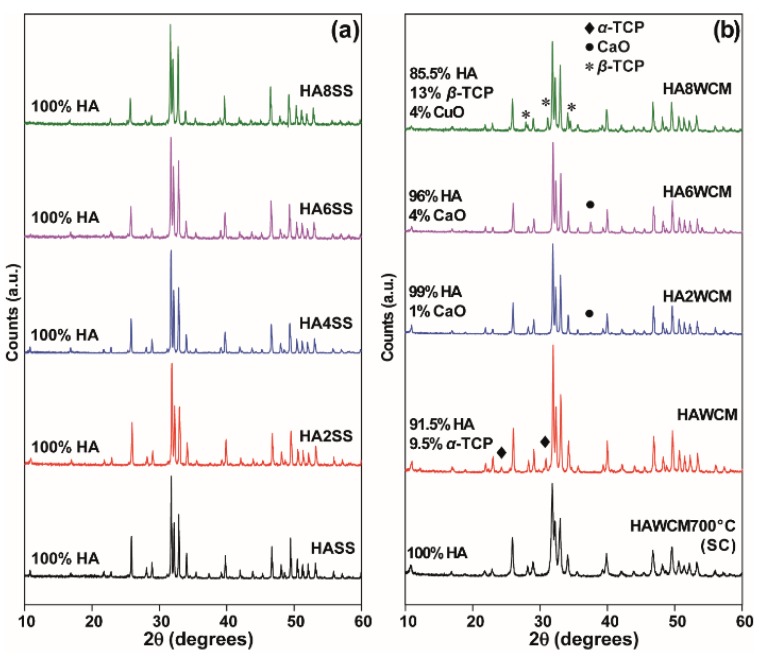
Powder X-ray diffraction (PXRD) data of the undoped and Cu-doped hydroxyapatite (HA) samples prepared by (**a**) solid-state reaction, and (**b**) the wet chemical synthetic routes.

**Figure 3:**
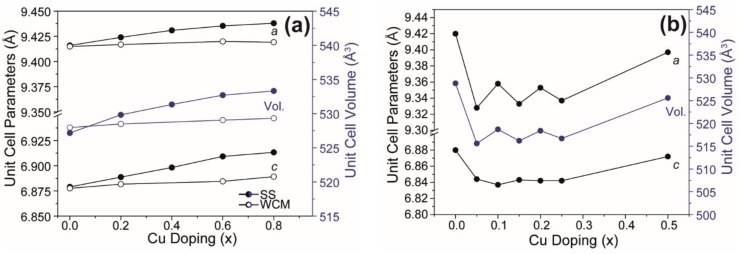
Unit cell and volume parameters (**a**) as determined from the PXRD for Ca_10_(PO_4_)_6_[Cu_x_(OH)_2-2x_O_x_] (0 ≤ x ≤ 0.8) generated by the solid-state and wet chemical synthesis methods in this work, compared with (**b**) the same parameters from Cu-doped systems generated by Shanmugam et al. [24].

The wet chemical route yielded single-phase Ca_10_(PO_4_)_6_(OH)_2_ HA after slow cooling from 700 °C but soaking at 1100 °C, and air quenching stabilised *α*-TCP as a secondary phase (~9.1 wt%), as reported previously (Figure 2b) [27]. For x = 0.2 and x = 0.6 in Ca_10_(PO_4_)_6_[Cu_x_(OH)_2-2x_O_x_] apatites, CaO appears in minor quantity, but for x = 0.8, *α*-TCP (tri-calcium phosphate) and *β*-TCP become significant. The solid-state materials developed secondary compounds after the fourth or fifth quench cycle (see Appendix A, Figure A1) as confirmed by quantitative Rietveld analysis (Figure 3a; solid circles). There is also a marked and consistent dilation of lattice parameters with increasing Cu content (Table 1) in contrast to the observations of Shanmugam and Gopal [24] who studied similar Cu-doped systems (see Figure 3b). For the present solid-state derived materials, unit cell expansion in the cupriferous analogues is primary evidence that Cu enters the HA channel X positions, rather than the Ca crystallographic sites, because direct substitution of Cu^2+^ (IR = 0.73 Å) for Ca^2+^ (IR = 1.00 Å) would lead to a contraction [1,10,11]. The corresponding wet chemical route analogues showed a less evident trend (Figure 3). A typical example is Ca_10_(PO_4_)_6_[Cu_0.2_(OH)_1.6_O_0.2_] (HA2WCM), which contains ~1 wt% CaO. Rietveld refinement indicated Cu in the channel (X) site, and a slight excess scattering from the Ca sites would be consistent with partial Cu incorporation and the appearance of CaO. However, this interpretation is not quantitative due to the correlation of site chemistry and site occupancy. Since the partitioning of Cu into the HA channel (X) and Ca (A^I^) positions perturbs the cell parameters in opposing fashions, the simultaneous operation of these mechanisms would lead to marginal overall changes to the unit cell character. Similarly, Ca_10_(PO_4_)_6_[Cu_0.6_(OH)_0.8_O_0.6_] (HA6WCM) shows a small volume change compared to the material produced by the solid-state method, and the ~4 wt% coexistence of CaO would be consistent with the increased displacement of Ca by Cu. The Rietveld refinements indicate excess scattering from the A sites that would be consistent with Cu incorporation.

### 3.2. Morphology

**Figure 4:**
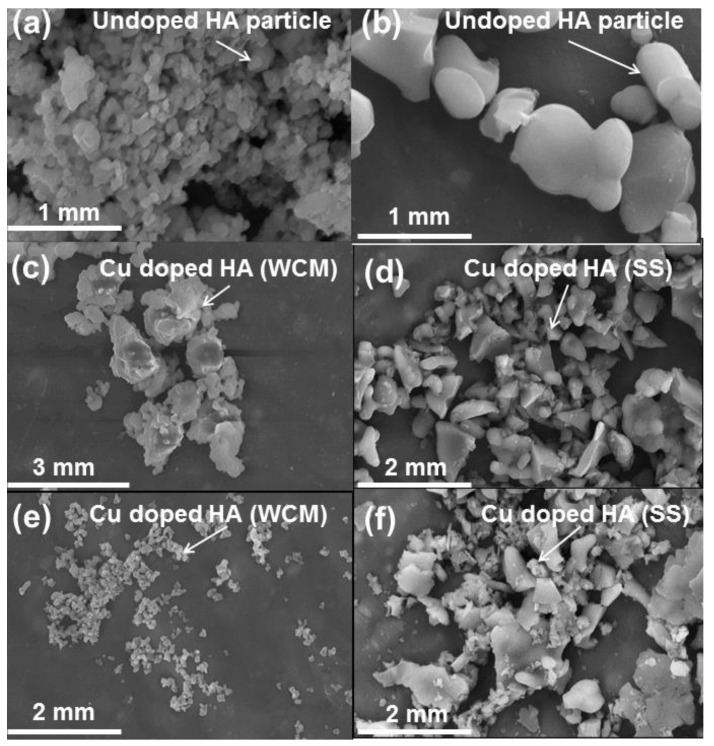
Field emission scanning electron microscopy (FESEM) images of HA and cupriferous HA systems, including (**a**) Ca_10_(PO_4_)_6_(OH)_2_ HAWCM, (**b**) Ca_10_(PO_4_)_6_(OH)_2_ HASS, (**c**) Ca_10_(PO_4_)_6_[Cu_0.6_(OH)_0.8_O_0.6_] HA6WCM, (**d**) Ca_10_(PO_4_)_6_[Cu_0.6_(OH)_0.8_O_0.6_] HA6SS, (**e**) Ca_10_(PO_4_)_6_[Cu_0.8_(OH)_0.4_O_0.8_] HA8WCM and (**f**) Ca_10_(PO_4_)_6_[Cu_0.8_(OH)_0.4_O_0.8_] HA8SS.

**Figure A2:**
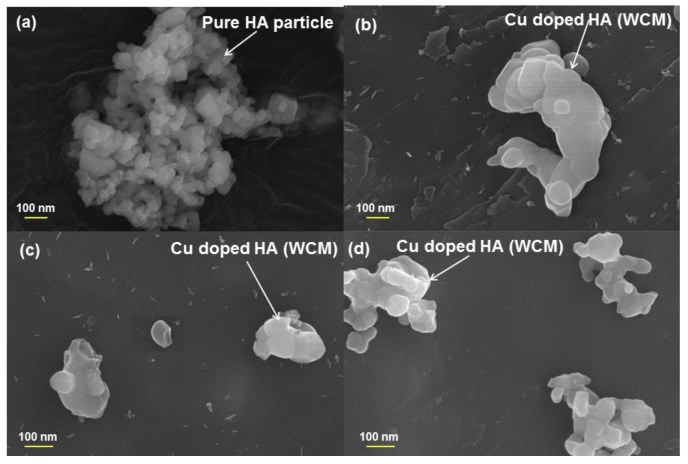
FESEM images of samples (**a**) HAWCM, (**b**) HA2WCM, (**c**) HA6WCM and (**d**) HA8WCM synthesised via the wet chemical route.

The undoped and cupriferous HA existed as agglomerates of varying size and morphology (see Figure 4). Apatites from wet chemistry precursors were irregular but became equant at higher Cu loadings (see Figure 4a,c,e and Appendix B, Figure A2) and showed more uniform particle sizes compared to the solid-state route (see Figure 4b,d,f) [31,32,33]. The larger apatite particles produced by solid-state processing could be seen from FESEM and was confirmed by laser scattering. These measurements showed that undoped HA prepared by the wet chemical route was characterised by an average particle size of 249 ± 66 nm, whereas the equivalent undoped HA prepared by the solid-state route was characterised by an average particle size of 1.09 ± 0.12 mm (approximately four times larger). Similarly, laser scattering measurements confirmed that cupriferous apatite particles produced by solid-state processing exhibited a larger average particle size of 1.74 ± 0.28 mm in comparison to particle sizes of 323 ± 42 nm produced by wet chemical processing (approximately five times larger). Furthermore, larger average particle sizes were exhibited by Cu-doped systems (in comparison to the undoped systems) irrespective of the preparative method. The solid-state materials show no correlation of morphology and Cu content (see Appendix B, Figure A2). Earlier studies demonstrated that the improved sorption capability of Cu-doped HA is attributable to a higher specific surface area [31,32].

### 3.3. Copper Oxidation State

**Figure 5:**
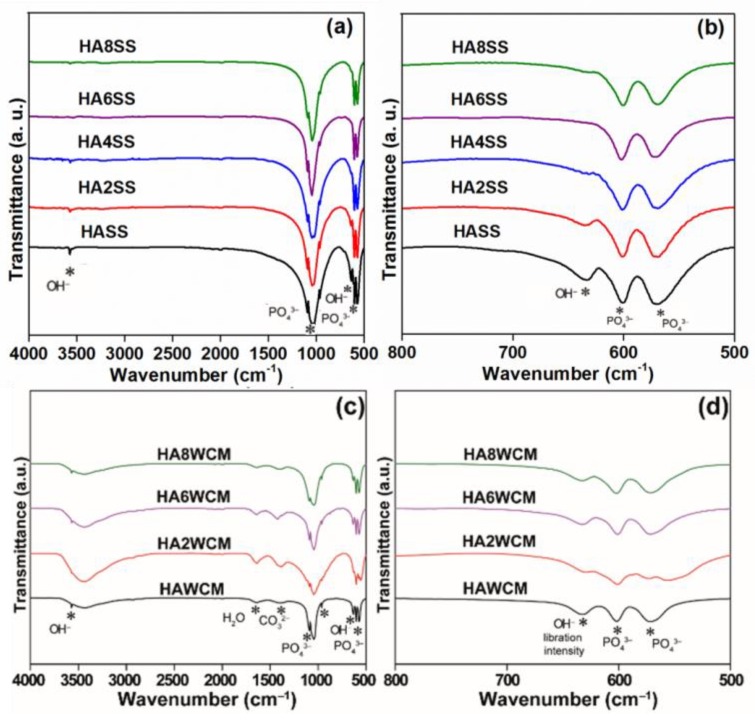
Fourier transform infrared (FTIR) spectra covering the ranges of 4000–500 cm^−1^ (**a**,**c**) and 800–500 cm^−1^ (**b**,**d**) for the solid-state synthesised apatites (**top**) and the wet chemical derived products (**bottom**).

Fourier transform infrared spectroscopy (FTIR) shows strong bands from 958–1097 cm^−1^ and 545–600 cm^−1^, attributable to symmetric and antisymmetric stretching vibrations and bending modes characteristic of the PO_4_^3−^ group with T_d_ symmetry (Figure 5a,d) [12,33,34]. The band centred at 3573 cm^−1^ is characteristic of hydroxyl group stretching (Figure 5a,c), while that at 630 cm^−1^ is ascribed to the OH^−^ libration (Figure 5b,d) [33]. For the solid-state derived apatites, the intensity of the OH^−^ libration band is observed to decrease monotonically with increasing Cu content and reaches a minimum for the HA8SS sample (see Figure 5b) consistent with the presumptive displacement of hydroxyl by oxygen in Ca_10_(PO_4_)_6_[Cu_x_(OH)_2-2x_O_x_]. This interpretation aligns with the unit cell volume increase (Table 1) and neutron diffraction confirmation that Cu^+^ replaces H^+^ in the channel [1]. Similarly, for Ca_10_(PO_4_)_6_[Cu_0.2_(OH)_1.6_O_0.2_] (HA2WCM) prepared by wet chemistry, the OH^−^ libration band intensity decreases with Cu doping, but for Ca_10_(PO_4_)_6_[Cu_0.6_(OH)_0.8_O_0.6_] (HA6WCM) and Ca_10_(PO_4_)_6_[Cu_0.8_(OH)_0.4_O_0.8_] (HA8WCM) it becomes more prominent. A broad band around the 2600–3713 cm^−1^ region and a weak band at approximately 1629 cm^−1^ for all samples confirm the presence of adsorbed water. In addition, a weak band around 1430 cm^−1^ arises from trace amounts of CO_3_^2−^, which may result from the reaction of atmospheric CO_2_ with Ca^2+^ and Cu^2+^ species in the surface layer of the apatite powders [33].

**Figure 6:**
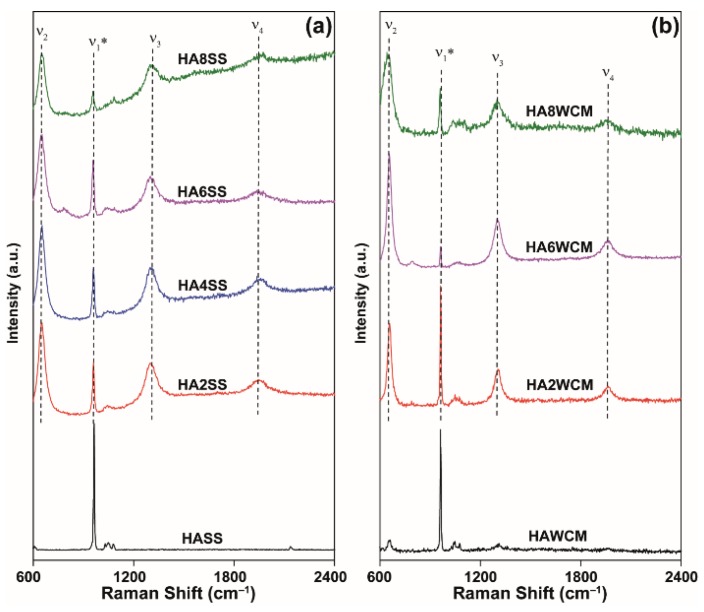
Raman spectra for cupriferous HA systems prepared by the (**a**) solid-state and (**b**) wet chemical synthesis routes.

The Raman spectra invariably show strong resonances from 650–655 cm^−1^ with two overtones at 1300–1308 cm^−1^ and 1950–1957 cm^−1^, respectively (see Figure 6a,b) [2,35,36]. Kazin et al. [35] reported similar bands in Cu-doped strontium hydroxyapatite and ascribed these to the O-Cu^3+^-O chromophore occupying the channel. However, the appearance of Cu^3+^ is unlikely in the present case [35]. The fully symmetrical stretching vibration ν_1_* of the PO_4_^3−^ groups can be assigned to the sharp band in the 961–963 cm^−1^ range [36], and the weak bands at approximately 1050 cm^−1^ are due to ν_3_ antisymmetric stretching vibrations bands [37].

**Figure 7:**
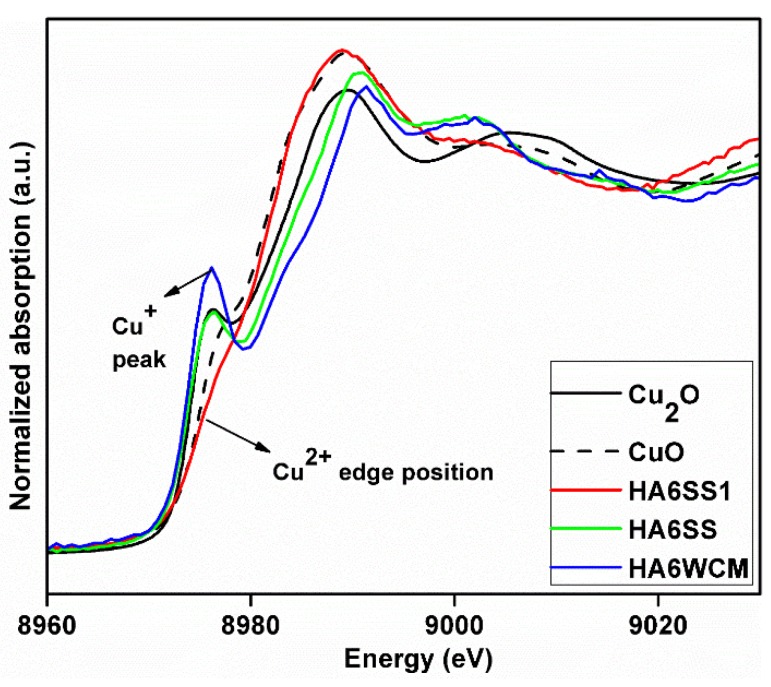
X-ray absorption spectra (XAS) data from x = 0.6 slow-cooled HA6SS1 sample compared to that derived from the air quenched H6SS and HA6WCM samples and Cu_2_O and CuO reference materials.

**Figure A3:**
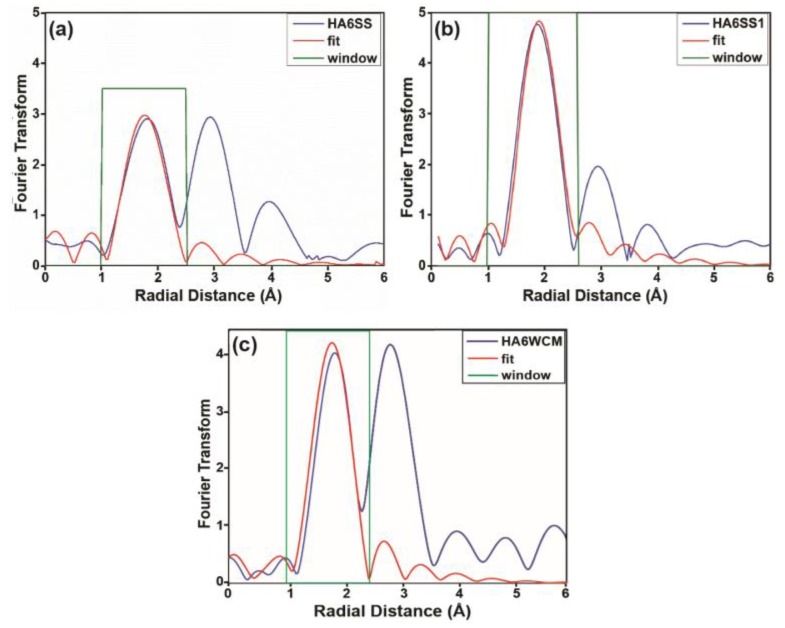
1st shell EXAFS data from (**a**) the air-quenched sample HA6SS obtained from the solid-state reaction route, (**b**) the slow cooled sample HA6SS1 obtained from the solid-state reaction route and (**c**) the air quenched sample HA6WCM obtained from wet chemical reaction route.

X-ray absorption spectroscopy (XAS) data of solid-state HA6SS and wet chemical HA6WCM samples (both air quenched from 1100 °C) were compared with x = 0.6 solid-state reacted HA6SS1 (slow cooled from 700 °C) and CuO (Cu^2+^) and Cu_2_O (Cu^+^) primary standards (see the X-ray absorption near edge structure (XANES) data of Figure 7). This study is inconclusive in proving that Cu^2+^ is present in the slow-cooled HA6SS1 sample, in contrast to Gomes et al. who reported a predominance of the Cu^2+^ species in cupriferous HA heat treated at 1100 °C [27]. In addition, the invariance of the measured Cu-O distances at ~1.8–1.9 Å measured from the extended X-ray absorption fine structure (EXAFS) data (see Appendix C, Figure A3) and the subsequent ^31^P MAS NMR study (see below) could not corroborate the presence of Cu^2+^ speciation in any of these systems.

### 3.4. Local Structure

**Figure 8:**
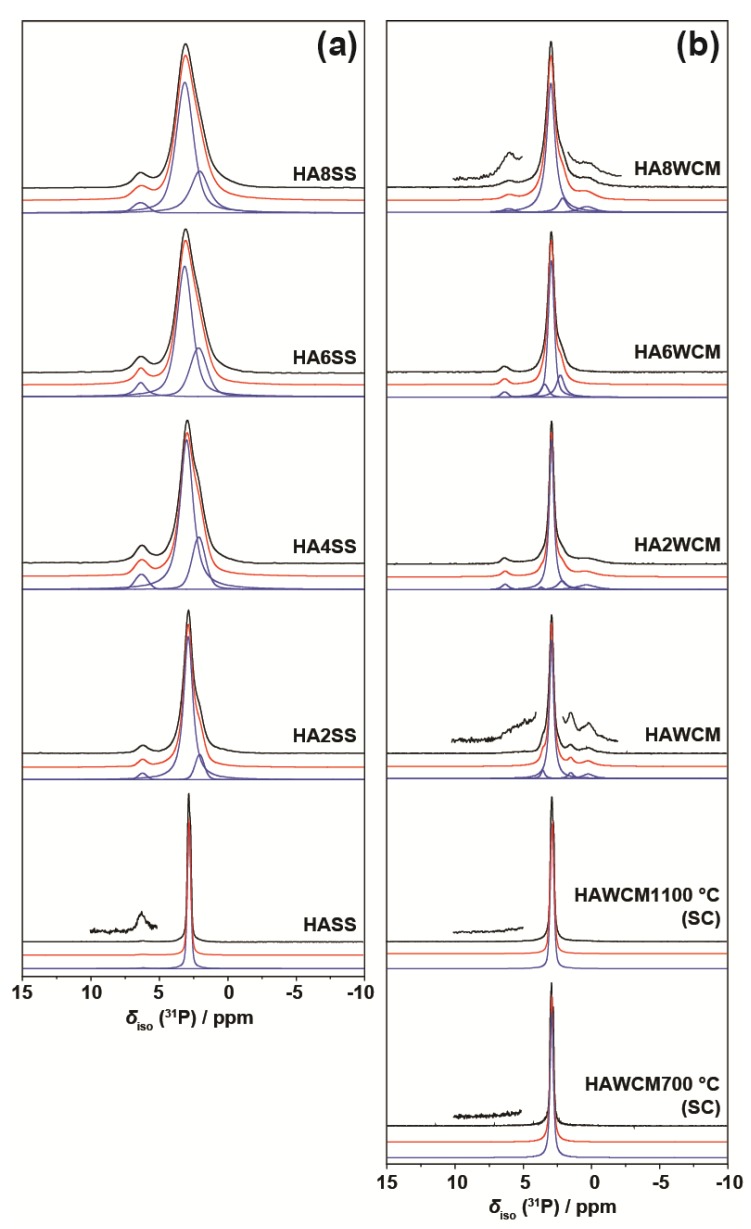
The ^31^P MAS NMR data (ν_0_(^31^P) = 242.96 MHz, ν_r_ = 12 kHz) showing the experimental spectra (black), total simulated spectra (red), and deconvoluted resonances using Gaussian line shapes (blue) from the suites of Cu-doped HA samples prepared by the (**a**) solid-state and (**b**) wet chemical synthetic routes.

**Figure 9:**
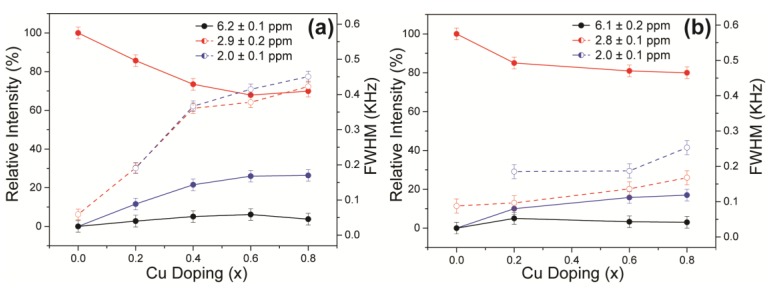
The quantitative estimates of the evolving P speciation with increasing Cu doping as determined by ^31^P MAS NMR measurements on the suites of Cu-doped HA samples prepared by the (**a**) solid-state and (**b**) wet chemical synthetic routes.

The ^31^P MAS NMR data acquired from the HA and Cu-doped HA systems synthesised by the solid-state and wet chemical methods are presented in Figure 8a,b, respectively. These data exhibit multiple resolved ^31^P resonances, suggesting that the complex P speciation in these Cu-doped biomaterials is dominated by at least three chemically distinct environments. All measured ^31^P chemical shifts and integrated intensities characterising these data are summarised in Table 2 and Figure 9a. The undoped Ca_10_(PO_4_)_6_(OH)_2_ (HASS) underpinning the solid-state synthesis series is dominated by a single narrow resonance at 2.7 ppm which is assigned to a well-formed hexagonal HA phase (see the bottom spectrum in Figure 8a). Upon Cu incorporation into this HA phase, two significantly broader resonances are now observed at ^31^P chemical shifts of ~3 ppm and ~2 ppm, which are assigned to the orthophosphate units comprising the perturbed hexagonal HA phase. The broader resonance at ~2 ppm is a consequence of the changing PO_4_ speciation characterising the Ca_10_(PO_4_)_6_[Cu_x_(OH)_2-2x_O_x_] system which is now influenced by positional disorder caused by the substitution of Cu^+^ metal ions into the [001] HA channel, as corroborated by the PXRD study above. This introduction of Cu^+^ into the [001] channel can introduce different PO_4_ species with marginally different chemical shifts from the parent material. Figure 3a shows that the lattice parameters and cell volume observed for the Cu-doped HA systems prepared via the solid-state route all exhibit a monotonic increase up to the maximum substitution value of x = 0.8. The direct quantitation of the P speciation elucidated from this ^31^P MAS NMR study depicted in Figure 9a exhibits similar monotonic trends describing the decrease of the species represented by the decrease in the ~3.0 ppm resonance and concomitant increase in the species represented by the ~2.0 ppm resonance. Furthermore, from Figure 9a and Table 2 it can be observed that the ^31^P resonance linewidths are constantly increasing throughout this substitutional series. These collective indicators suggest that increasing short range positional disorder is associated with progressive Cu incorporation.

A less intense third resonance is also observed at a chemical shift of ~6.0 ppm which is assigned to distorted PO_4_ environments proximate to channel vacancies. This ^31^P chemical shift has been previously identified from thermal decomposition studies of HA systems, and these structural vacancies have also been shown to form readily under more rapid quenching conditions [38,39]. A very small trace of this species is present in the undoped HA sample from the solid-state synthesis suite (see the vertically expanded inset on the HASS data, Figure 8a), and it is postulated that this initial vacancy formation helps to initiate the Cu incorporation process. It is interesting to note from Figure 9a that, within experimental error, the concentration of this species is largely unchanged throughout this Cu dopant series. In similar fashion to the XAS and Raman studies reported above, this ^31^P MAS NMR study cannot detect any paramagnetic influence upon the reported shifts or linewidths that can be associated with the presence of Cu^2+^ species.

For the analogous ^31^P MAS NMR data measured from wet chemical synthesis series (see Figure 8b and Table 2), this suite of samples is underpinned by the well-formed Ca_10_(PO_4_)_6_(OH)_2_ phases HAWCM 700 °C and HAWCM 1100 °C produced from slow-cooled preparations. Like the HASS sample from the air-quenched solid-state syntheses, these samples also exhibit a dominant resonance at 2.7 ppm; however, for these latter cases, there is no evidence indicating the formation of channel vacancies as indicated by the ^31^P resonance at ~6 ppm. Furthermore, the slow cooling process adopted for samples HAWCM 700 °C and HAWCM1100°C has also avoided the onset of α-TCP crystallisation. The undoped HA sample produced from the air quenched wet chemical method (HAWCM) does exhibit some resonance intensity at ~6 ppm, although the actual contribution from channel vacancies is partially obscured by the manifold of 12 ^31^P resonances (observed over the range of ~−1–5 ppm) that characterises the formation of *α*-TCP [40,41]. This observation of *α*-TCP formation is corroborated by the PXRD data in Figure 2b. Additional broad upfield resonances at ~0 ppm are also observed in the air-quenched wet chemical sample HA8WCM, and these are assigned to *β*-TCP in agreement with the PXRD data in Figure 2b [42,43]. A direct comparison of Figure 8a,b indicates that the wet chemical synthesised suite of samples exhibits a distinct reduction in intensity of the ~6 ppm resonance associated with structural vacancies in comparison to the solid-state synthesised suite. More importantly, the variation in the quantitative analyses of the P species represented by resonances at ~3 ppm and ~2 ppm is approximately half of the total variation exhibited by the solid-state prepared samples (see Table 2 and the Figure 9b), and the ^31^P MAS NMR linewidths and linewidth variation is much less pronounced (also approximately half of that exhibited by the solid-state prepared samples). These observations are corroborated by the significantly reduced variation of the lattice parameter and cell volume parameters represented in Figure 3a, and thus infer that the levels of Cu incorporation into the HA structure via the wet chemical methods are around half that achieved with the solid-state syntheses.

### 3.5. Antimicrobial Efficacy

**Figure 10:**
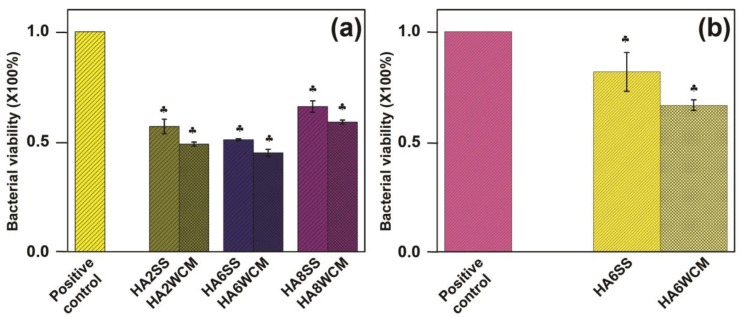
Antibacterial assay of the synthesised Cu-doped HA samples against (**a**) gram-negative bacteria (*E. coli*) and (**b**) gram-positive bacteria (*S. aureus*).

All Cu-doped HA were effective against *E. coli* with two tailed p values less than 0.0001 with significantly enhanced antibacterial efficiency in passing from 35% (Ca_10_(PO_4_)_6_[Cu_0.8_(OH)_0.4_O_0.8_] (HA8SS) to 55% Ca_10_(PO_4_)_6_[Cu_0.6_(OH)_0.8_O_0.6_] (HA6WCM) (Figure 10a). For both preparative techniques, the x = 0.6 sample Ca_10_(PO_4_)_6_[Cu_0.6_(OH)_0.8_O_0.6_] showed the best antibacterial efficacy. To test the broader potential of cupriferous HA as an antibacterial agent, HA6WCM and HA6SS (the best performing compositions against *E. coli*) were tested against *S. aureus*, and showed 25% and 40% antibacterial efficiency, respectively (Figure 10b). Shanmugam and Gopal reported that the antibacterial efficacy of Cu-doped HA varies from 25% to 85%, where it was assumed that the Cu substituted at the Ca site, although this was not confirmed [24]. Differences in lattice parameter response with increasing Cu content in the present study compared to that of Shanmugam and Gopal [24] (see Figure 3a,b) suggest that a direct comparison between these studies is unwarranted. In all instances, the efficacy of the apatites derived by the wet chemical route was superior to solid-state materials with the same copper content. This is expected as the smaller particle size and larger surface area would expose a greater proportion of the bacteria to copper-rich surfaces.

While Cu^+^ is reported as a more effective antibacterial agent compared to Cu^2+^ [44,45], it is not confirmed that divalent Cu is present in the materials studied here. For the same nominal composition, wet chemical route Cu-doped apatites have improved antibacterial properties in comparison to the solid-state synthesised samples due to the finer particle size (~500 nm) and near spherical shape. Finer particle sizes present a greater surface area for the Cu ions to interact with the bacterial cells. Alternatively, the improved performance of the wet chemical route materials could be due to partial Cu substitution at the Ca sites, which is reported to give a better antibacterial efficiency, but further work is required. Kim et al. [46] suggested that the use of Cu^+^ nitrate salt as a precursor might not show an antibacterial effect against *E. coli* due to the presence of “nitrate” apatite, although this conclusion is not supported by the present work.

## 4. Conclusions

Cupriferous HA Ca_10_(PO_4_)_6_[Cu_x_(OH)_2-2x_O_x_] (0.0 ≤ x ≤ 0.8) was prepared by conventional solid-state reaction and a wet chemical precipitation routes. The PXRD and the ^31^P MAS NMR studies revealed that the substitution site of Cu into the HA framework is controlled by (primarily) the synthesis method and heat treatment process, with the solid-state route yielding single phase HA across all Cu concentrations (x ≤ 0.6). The wet chemical materials were often multiphase but show superior antibacterial activity against *E. coli* and *S. aureus* (~20% improvement) due to the finer particle size and greater surface area for bacterial interaction. In addition, the wet chemical route may allow partial Cu replacement of Ca, rather than in the apatite channel. This may lead to improved antimicrobial efficacy. The XAS, Raman and solid-state NMR studies confirm that Cu^+^ is the dominate oxidation state within these suites of prepared samples, with no definitive evidence for the presence of Cu^2+^ able to be measured. Previous antibacterial characterisation studies have shown that Cu+ is more toxic to bacteria as compared to Cu^2+^ [44,45]. Antibacterial efficacy of Cu sited in the HA channel (X) position is a new finding, and the effect of mixed Cu oxidation states is worthy of further investigation. Future work could be directed to quantifying the efficiency of multi-valent Cu-doped HA against alternative microbe communities.

## Figures and Tables

**Table 1:** Sample ID, nominal compositions and lattice parameters of cupriferous hydroxyapatites.

Synthesis	Sample ID	Nominal Composition	x Nominal	*a* (Å)	*c* (Å)	V (Å^3^)
**Solid State Method**	HASS*^a^*	Ca_10_(PO_4_)_6_(OH)_2_	0	9.4159(3)	6.8790(3)	527.17(4)
	HA2SS*^a^*	Ca_10_(PO_4_)_6_[Cu_0.2_(OH)_1.6_O_0.2_]	0.2	9.4240(4)	6.8887(3)	529.82(5)
	HA4SS*^a^*	Ca_10_(PO_4_)_6_[Cu_0.4_(OH)_1.2_O_0.4_]	0.4	9.4309(1)	6.8983(1)	531.34(2)
	HA6SS*^a^*	Ca_10_(PO_4_)_6_[Cu_0.6_(OH)_0.8_O_0.6_]	0.6	9.4353(2)	6.9093(2)	532.70(3)
	HA6SS1*^b^*	Ca_10_(PO_4_)_6_[Cu_0.6_(OH)_0.8_O_0.6_]	0.6	-	-	-
	HA8SS*^a^*	Ca_10_(PO_4_)_6_[Cu_0.8_(OH)_0.4_O_0.8_]	0.8	9.4380(3)	6.9133(2)	533.31(4)
**Wet Chemical Method**	HAWCM700°C*^b^*	Ca_10_(PO_4_)_6_(OH)_2_	0	9.4149(6)	6.8776(5)	527.96(8)
	HAWCM1100°C*^b^*	Ca_10_(PO_4_)_6_(OH)_2_	0	9.4149(6)	6.8776(5)	527.96(8)
	HAWCM*^a^*	Ca_10_(PO_4_)_6_(OH)_2_	0	9.4168(3)	6.8792(3)	528.30(5)
	HA2WCM*^a^*	Ca_10_(PO_4_)_6_[Cu_0.2_(OH)_1.6_O_0.2_]	0.2	9.4168(3)	6.8817(3)	528.49(4)
	HA6WCM*^a^*	Ca_10_(PO_4_)_6_[Cu_0.6_(OH)_0.8_O_0.6_]	0.6	9.4199(3)	6.8845(3)	529.05(4)
	HA8WCM*^a^*	Ca_10_(PO_4_)_6_[Cu_0.8_(OH)_0.4_O_0.8_]	0.8	9.4191(3)	6.8892(3)	529.32(5)

*^a^* air quenched from 1100 °C. *^b^* slow cooled from 700 °C.

**Table 2:** ^31^P MAS NMR chemical shift, linewidth and relative intensity parameters determined from the solid-state and wet chemical synthesised Cu-doped HA systems (Ca_10_(PO_4_)_6_[Cu_x_(OH)_2-2x_O_x_].

Synthesis	Sample ID	Nominal Composition	x Nominal	*δ* (^31^P) (ppm) (±0.1)	FWHM (kHz) (±0.01)	Relative Intensity*^c^* (kHz) (±2)
**Solid-State Method**	HASS*^a^*	Ca_10_(PO_4_)_6_(OH)_2_	0	6.0	0.11	1
				2.7	0.06	99
	HA2SS*^a^*	Ca_10_(PO_4_)_6_[Cu_0.2_(OH)_1.6_O_0.2_]	0.2	6.2	0.28	3
				2.9	0.24	83
				2.1	0.21	14
	HA4SS*^a^*	Ca_10_(PO_4_)_6_[Cu_0.4_(OH)_1.2_O_0.4_]	0.4	6.3	0.29	6
				3.0	0.33	65
				2.1	0.34	30
	HA6SS*^a^*	Ca_10_(PO_4_)_6_[Cu_0.6_(OH)_0.8_O_0.6_]	0.6	6.2	0.31	5
				3.0	0.38	67
				2.0	0.40	28
	HA8SS*^a^*	Ca_10_(PO_4_)_6_[Cu_0.8_(OH)_0.4_O_0.8_]	0.8	6.2	0.31	6
				3.0	0.42	67
				1.9	0.45	27
**Wet Chemical Method**	HAWCM700°C*^b^*	Ca_10_(PO_4_)_6_(OH)_2_	0	2.7	0.04	100
	HAWCM1100°C*^b^*	Ca_10_(PO_4_)_6_(OH)_2_	0	2.7	0.04	100
	HA2WCM*^a^*	Ca_10_(PO_4_)_6_[Cu_0.2_(OH)_1.6_O_0.2_]	0.2	6.2	0.15	5
				2.8/3.0	0.10	85
				2.0	0.19	10
				0.2	0.41	-
	HA6WCM*^a^*	Ca_10_(PO_4_)_6_[Cu_0.6_(OH)_0.8_O_0.6_]	0.6	6.2	0.24	3
				2.8/3.0	0.11	81
				2.1	0.49	16
	HA8WCM*^a^*	Ca_10_(PO_4_)_6_[Cu_0.8_(OH)_0.4_O_0.8_]	0.8	5.9	0.18	3.0
				2.9/3.1	0.17	80.0
				2.1	0.21	17
				0.3	0.26	-

^*a*^ air quenched from 1100 °C. ^*b*^ slow cooled from 700 °C. ^*c*^ integrated intensity calculations only include P species within each Cu-doped HA system (impurity phases not included).

## Appendix A

As discussed in the main text, the preparation of single-phase Cu-doped HA via the solid-state synthesis route is only achieved after the 4th or 5th quenching cycle, as shown for case of sample HA2SS below. In contrast, a similar result is achieved after one only quench cycle when the wet chemical method is implemented. This is the primary evidence suggesting that the wet chemical approach is more time efficient approach for producing single phase Cu-doped HA systems.

## Appendix B

The main text discussion reports that a particle size <500 nm is observed from the FESEM images of the samples produced via the wet chemical route. This smaller particle size of wet chemical route samples shows an improved antibacterial efficacy compared to the solid-state prepared samples.

## Appendix C

As pointed out in the main text, the EXAFS data of certain air quenched and slow cooled samples prepared via the solid-state and wet chemical routes are presented below. The first shell EXAFS fittings were used to determine the Cu-O distances in the respective samples. These distances all appear invariant within a ~1.8–1.9 Å range, thus suggesting the presence of a constant Cu oxidation state (i.e., Cu^+^) throughout.
